# Supplementary material for: Magnetic neutron scattering by magnetic vortices in thin submicron-sized soft ferromagnetic cylinders
Source: Sci Rep. 2016 Apr 26;6:25055. doi: 10.1038/srep25055 (PMC4844968; doi:10.1038/srep25055)
Supplement: Supplementary Information [file srep25055-s1.pdf]

Magnetic neutron scattering by magnetic vortices in thin submicron-sized soft ferromagnetic cylinders

Konstantin L. Metlov[1,*] **and Andreas Michels**[2,+]

[1]Donetsk Institute for Physics and Technology, Donetsk, 83114, Ukraine
[2]University of Luxembourg, Physics and Materials Science Research Unit, Luxembourg, L-1511, Grand Duchy of Luxembourg
[*]metlov@fti.dn.ua
[+]Andreas.Michels@uni.lu

ABSTRACT

In this Supplemental Material, we provide a sketch of the scattering geometry and expressions for the magnetization Fourier components of a displaced magnetic vortex.

Sketch of the perpendicular scattering geometry

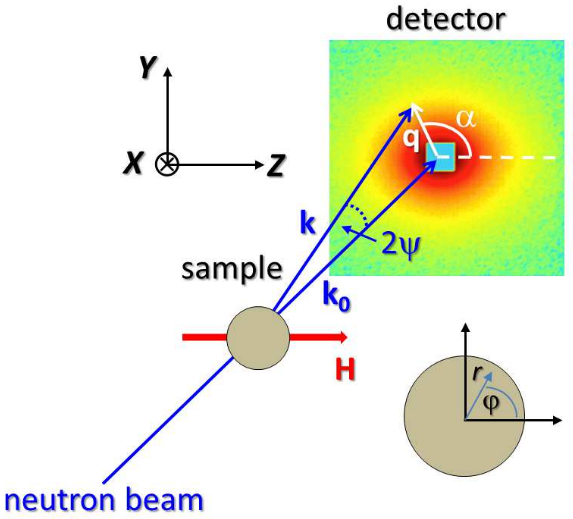

Figure 1. Sketch of the perpendicular scattering geometry which has the applied magnetic field $\mathbf{H}$ perpendicular to the wave vector $\mathbf{k}_0$ of the incident neutron beam; $q = |\mathbf{q}| = 4\pi\lambda^{-1}\sin\psi$, where 2ψ denotes the scattering angle and λ is the mean neutron wavelength. Note that $\mathbf{H} \parallel \mathbf{Z}$ and that in the small-angle approximation $\mathbf{q} \cong q(0, q_y, q_z) = q(0, \sin\alpha, \cos\alpha)$.

Magnetization Fourier components of a displaced magnetic vortex

The parallel and perpendicular SANS cross sections are customarily expressed in a coordinate system where the neutrons travel, respectively, along the Z and X-axis, but the direction of the applied magnetic field is always along the Z-direction. Let us express the magnetization Fourier components in the coordinate system corresponding to the perpendicular geometry (see Fig 1 above), noting that in the parallel geometry the magnetic vortex is not displaced ($b = 0$).

The Fourier transform of the magnetization in the vortex state [Eq. (9)] up to the first order in b (valid in the low-field linear part of the hysteresis loop in Fig. 2) can be expressed as:

$$\widetilde{M}_i = M_\mathrm{S} L R^2 \left(\widetilde{\mu}_i^0 + b\widetilde{\mu}_i^1 + O(b^2)\right) \frac{1}{\sqrt{2\pi}} \frac{\sin(Lq_\mathrm{X}/2)}{Lq_\mathrm{X}/2}$$

with $i = \mathrm{X,Y,Z}$ being the Cartesian coordinate-system axis labels, and the dimensionless quantities $\widetilde{\mu}$ being:

$$\begin{aligned}
\{\widetilde{\mu}^0_{\mathrm{Z}}, \widetilde{\mu}^0_{\mathrm{Y}}\} &= \imath\,\widetilde{\mu}^0_{\perp}\{\sin\alpha, -\cos\alpha\},\\
\widetilde{\mu}^0_{\perp} &= \frac{2p^2 F_1(\gamma k)}{c^2} + \frac{F_2(k) - F_2(\gamma k)}{k^2}\\
\widetilde{\mu}^0_{\mathrm{X}} &= \widetilde{\mu}^0_{\parallel} = \gamma^2 G_1(\gamma k),
\end{aligned}$$

where $k = q_\perp R$, $\gamma = p/c$ and the vector $\boldsymbol{q}_\perp = \{q_{\mathrm{Z}}, q_{\mathrm{Y}}\} = q_\perp\{\cos\alpha, \sin\alpha\}$ is represented by its polar coordinates $\{q_\perp, \alpha\}$. The first-order terms are less symmetric:

$$\begin{aligned}
\widetilde{\mu}^1_{\mathrm{Z}} &= \frac{1}{p}\Big(2\gamma^3\left(c^2F_3(\gamma k) - F_4(\gamma k) + (1-c^2)\cos 2\alpha F_5(\gamma k)\right) +\\
&\frac{1}{k^3}\Big(\cos^2\alpha\left(F_6(\gamma k) - F_6(k) + k^2p^2\left(F_7(k) - F_7(\gamma k)\right)\right) +\\
&\cos 2\alpha\left(F_2(k) - F_2(\gamma k) + k^2p^2\left(F_8(\gamma k) - F_8(k)\right)\right)\Big)\Big),\\
\widetilde{\mu}^1_{\mathrm{Y}} &= \sin 2\alpha\Big(\frac{p}{2k}\left(F_{10}(\gamma k) - F_{10}(k)\right) +\\
&\frac{2(1-c^2)p^2}{c^3}F_5(\gamma k) + \frac{1}{2pk^3}\left(F_9(k) - F_9(\gamma k)\right)\Big),\\
\widetilde{\mu}^1_{\mathrm{X}} &= -\frac{4\imath p^2}{c^3}\left(c^2G_2(\gamma k) + G_3(\gamma k)\right)\sin\alpha.
\end{aligned}$$

The terms proportional to $F_i(\gamma k)$ and $G_i(\gamma k)$ correspond to the vortex core. Their contribution vanishes when the vortex core is neglected by taking the limit $c \to \infty$. The rest of the terms, proportional to $F_i(k)$, correspond to the meron part of the magnetization distribution.

The special functions $F_j(x)$ and $G_j(x)$ are defined as follows:

$$\begin{aligned}
F_1(x) &= \int_0^1 \frac{\rho^2 J_1(x\rho)}{1+\rho^2}\,\mathrm{d}\rho, \qquad F_2(x) = \int_0^x \rho J_1(\rho)\,\mathrm{d}\rho,\\
F_3(x) &= \int_0^1 \frac{\rho J_0(x\rho)}{(1+\rho^2)^2}\,\mathrm{d}\rho, \qquad F_4(x) = \int_0^1 \frac{\rho^5 J_0(x\rho)}{(1+\rho^2)^2}\,\mathrm{d}\rho,\\
F_5(x) &= \int_0^1 \frac{\rho^3 J_2(x\rho)}{(1+\rho^2)^2}\,\mathrm{d}\rho, \qquad F_6(x) = \int_0^x \rho^2 J_0(\rho)\,\mathrm{d}\rho,\\
F_7(x) &= \int_0^x J_0(\rho)\,\mathrm{d}\rho, \qquad F_8(x) = \int_0^x (J_1(\rho)/\rho)\,\mathrm{d}\rho,\\
F_9(x) &= \int_0^x \rho^2 J_2(\rho)\,\mathrm{d}\rho, \qquad F_{10}(x) = \int_0^x J_2(\rho)\,\mathrm{d}\rho,\\
G_1(x) &= \int_0^1 \frac{\rho(1-\rho^2)J_0(x\rho)}{1+\rho^2}\,\mathrm{d}\rho,\\
G_2(x) &= \int_0^1 \frac{\rho^2 J_1(x\rho)}{(1+\rho^2)^2}\,\mathrm{d}\rho, \qquad G_3(x) = \int_0^1 \frac{\rho^4 J_1(x\rho)}{(1+\rho^2)^2}\,\mathrm{d}\rho.
\end{aligned}$$

Plots of the $\widetilde{\mu}$ functions are shown in Fig. 2.

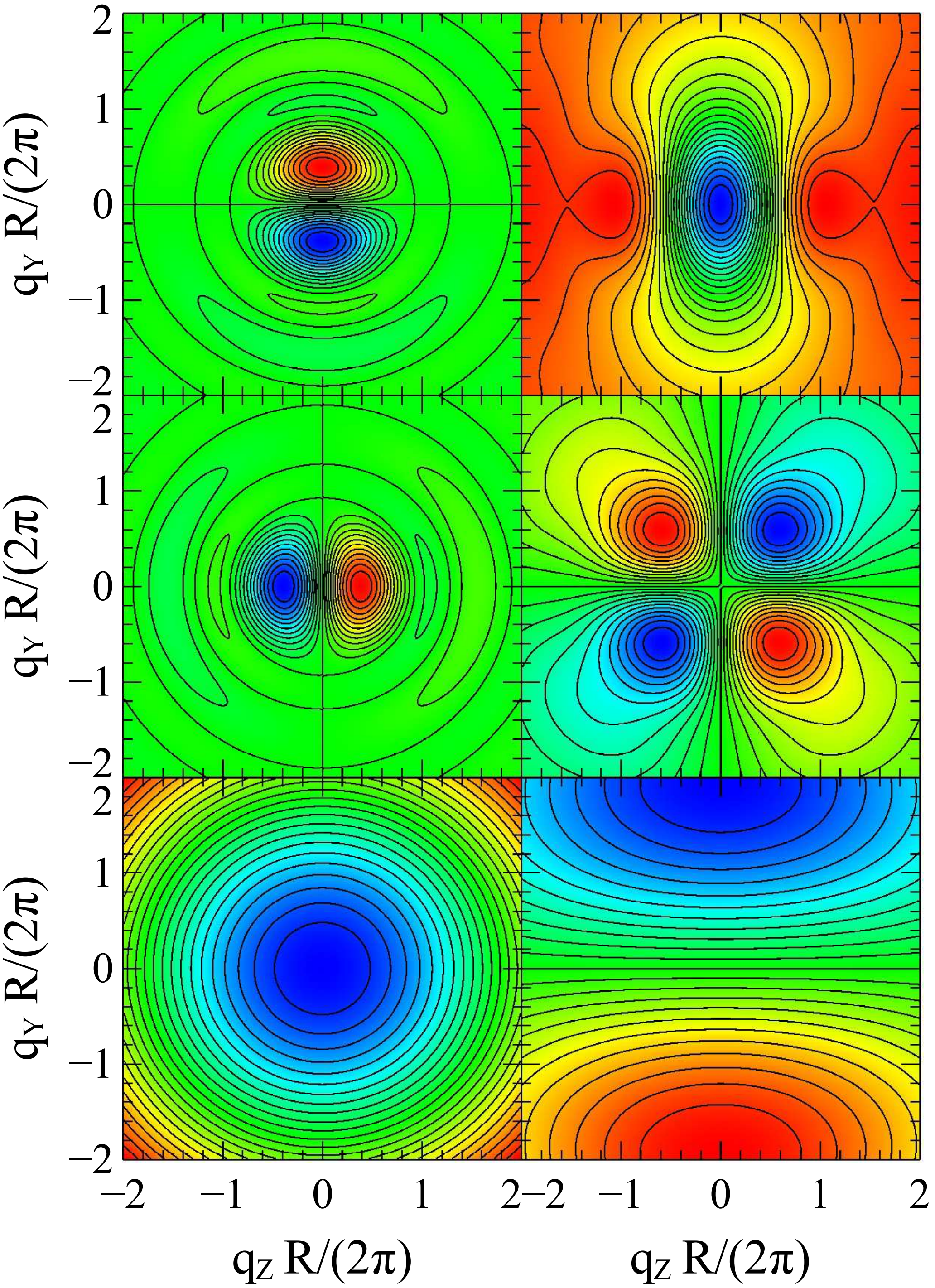

Figure 2. Fourier representation of the magnetization components in the vortex state [Eq. (9)] in the $q_Z - q_Y$ plane for $p = 1$, $c = 2$, and $q_X = 0$. The plots from top to bottom show $\widetilde{\mu}_Z$, $\widetilde{\mu}_Y$, $\widetilde{\mu}_X$. Left half corresponds to the centered vortex $\widetilde{\mu}^0$, right half shows the first-order terms $\widetilde{\mu}^1$ with respect to the vortex-center displacement b. Since the Fourier components are either purely real or purely imaginary complex numbers, their real or imaginary parts in either of these cases is plotted.
